# Antimicrobial efficacy of 45S5 and borosilicate bioglasses against multidrug-resistant bacteria: an *in vitro* assay

**DOI:** 10.1007/s42770-026-01936-6

**Published:** 2026-05-13

**Authors:** Mariana Bugov, Mareliza Possa de Menezes, Romário Alves Rodrigues, Gabriel Luiz Montanhim, Herlem Camila Pinto da Silva, Leandro Fernandes, Marita Vedovelli Cardozo, Paola Castro Moraes

**Affiliations:** 1 Department of Veterinary Clinic and Surgery, School of Agricultural and Veterinary Sciences, São Paulo State University (UNESP), Jaboticabal, SP Brazil; 2 Department of Pathology Reproduction and One Health, School of Agricultural and Veterinary Sciences, São Paulo State University (UNESP), Jaboticabal, SP Brazil; 3 Department of Dental Materials and Prosthetics, School of Dentistry of Araraquara, São Paulo State University (UNESP), Jaboticabal, Brasil

**Keywords:** Antimicrobial activity, Biofilms, Bioceramics, Bioglass, Hydroapatite, Bone repair

## Abstract

Several bioceramics have been developed to promote bone repair. However, postoperative infections in the implanted area may led to in bone resorption. The widespread use of antimicrobials has contributed to the increased prevalence of multidrug-resistant bacteria, posing an additional challenge to therapeutics. This study aimed to evaluate the antimicrobial activity and resistance-inducing potential of two bioceramic formulations, 45S5 bioglass and borosilicate bioglass (BVBS), against multidrug-resistant strains of methicillin-resistant *Staphylococcus pseudintermedius* (MRSP), *Escherichia coli*, and *Pseudomonas aeruginosa*. Antibacterial activity was determined by the minimum inhibitory concentration (MIC) and time-kill kinetics. Resistance-inducing capacity was assessed by bacterial growth after 48 h of exposure to 800 mg/mL of each bioceramic. Bioglass 45S5 demonstrated greater antimicrobial activity, with an MIC of 50 mg/mL for *E. coli* and *P. aeruginosa* and 100 mg/mL for MRSP, and complete eradication of the strains after 8 h. BVBS presented an MIC of 100 mg/mL for *E. coli* and *P. aeruginosa*, and 200 mg/mL for MRSP, with complete bacterial elimination within 24 h. No resistance induction was observed for 45S5 in any of the strains, and BVBS showed this effect only on *E. coli* and *P. aeruginosa* strains. The results indicate that both formulations were effective against multidrug-resistant bacteria, with bioglass 45S5 being the most promising alternative due to its greater antimicrobial efficacy and lack of resistance induction.

## Background

Currently, the use of synthetic materials in routine veterinary surgery is increasing, particularly as bone substitutes capable of stimulating the repair process more rapidly and effectively. These biomaterials are indicated for a wide range of injuries that cause significant bone loss, such as those resulting from trauma, infections, and bone neoplasms, with the goal of promoting tissue remodeling and reconstruction [1–3]. Among these materials, biocompatible ceramic composites stand out, produced in situ or in vivo through various chemical processes. They exhibit excellent biological integration properties due to their structural similarity to natural hydroxyapatite [4]. When in contact with bone, the mineral hydroxyapatite acts as an osteoconductive agent, promoting new bone formation at the implant interface [5, 6]. In recent years, interest in the potential antibacterial properties of bioceramics has increased. Several mechanisms of action have been proposed, including changes in local pH, increased osmotic pressure, and the release of glass fragments capable of damaging bacterial cell walls, facilitating the entry of antimicrobial agents into the bacterial cytoplasm [7].

Several bioceramic formulations have already been approved by the United States *Food and Drug Administration* (FDA) and are currently available on the market [7]. Among them, 45S5 bioglass stands out as a silica-based compound with a high capacity for bonding to bone tissue, trigged by its interaction with body fluids, which leads to the formation of a hydroxyapatite-like layer on its surface. This interaction stimulates bone growth at the bone-implant interface, favoring the integration process [8]. Boron, in turn, is an essential element for homeostasis. It is present in the composition of bones, nails, hair, and teeth, and is essential for maintaining bone health by participating in the regulation of calcium, magnesium, and phosphorus absorption [9]. Recently, boron has been incorporated into 45S5 bioglass, improving its mechanical properties. This modification enhances the material’s biological activity, particularly in relation to the action of phosphates and their susceptibility to hydrolysis, favored by the formation of borohydroxyl bonds (B – OH) in the presence of water, which catalyzes the bioactivity of the derivatives [10].

Despite their recognized osteoinductive potential, the use of bioceramics as orthopedic implants may be associated with the development of surgical site infections (SSI), which compromises the success of the procedure. Microbial colonization and biofilm formation on the surface of the biomaterial may contribute to implant failure/loosening and bone loss [11]. Postoperative osteomyelitis is characterized by bone loss secondary to a septic process, accompanied by local inflammatory response and, in many cases, requiring surgical reintervention and debridement [12]. Furthermore, the presence of multidrug-resistant bacteria poses a therapeutic challenge, limiting treatment options and increasing patient morbidity [13].

Given this context, evaluating the antimicrobial activity of bioceramics becomes essential for developing more effective strategies for preventing and treating infections caused by multidrug-resistant microorganisms in veterinary patients. Therefore, the present study aims to investigate the antimicrobial activity of 45S5 and BVBS bioglasses against multidrug-resistant bacterial strains, contributing to the advancement of knowledge about their therapeutic potential and clinical applicability.

## Methods

### Bacterial strains

To evaluate antimicrobial activity, three single clinical isolates (one per species), classified as multidrug-resistant and obtained from clinical infections, were used, named: *Escherichia coli*,* Pseudomonas aeruginosa*, and methicillin-resistant *Staphylococcus pseudintermedius* (MRSP). All three strains were previously isolated and phenotypically characterized in the Microbiology Laboratory of São Paulo State University “Júlio de Mesquita Filho” (UNESP), Jaboticabal campus, São Paulo, according to the guidelines established by the *Clinical and Laboratory Standards Institute* (CLSI, 2023). Antimicrobial susceptibility testing was performed using the agar disk diffusion method, with results interpreted based on the breakpoints recommended by CLSI.

### Bioceramics

Two distinct bioglass formulations were used: 45S5 bioglass and BVBS bioglass. Both were developed and produced in collaboration with the Department of Dental Materials and Prosthetics of the Araraquara School of Dentistry (São Paulo State University-UNESP) and the University of Araraquara (UNIARA), Araraquara, São Paulo, Brazil. The materials were supplied in powder form and properly sterilized prior to in vitro testing. Bioglass production followed the protocol described by Furlan [14], with NaCl excluded from the original formulation (Table 1). These modifications aimed to improve the physicochemical properties and reduce the cytotoxicity of the tested biomaterials. For the BVBS formulation, 2.5% (w/w) boric acid was incorporated into the composition.

**Table 1:** Proportion between components for the formation of bioglass

Component	Calculated as	Mole percentage (%)	Mass percentage (%)
30% colloidal silica	SiO2	45	41,93
Sodium oxide	Na2O	24,5	23,55
Calcium oxide	CaO	24,5	21,31
Phosphorus oxide	P2O5	6	13,21

### pH determination

The bioglass formulations were incubated in Brain Heart Infusion (BHI) broth at concentrations ranging from 10 to 400 mg/mL and maintained under continuous agitation (100 rpm) at 37 °C for 48 h under aerobic conditions. Measurements of pH were performed using a previously calibrated digital pHmeter at the following time intervals: 2, 4, 6, 8, 24, and 48 h hours. These time points were selected to correspond to the main microbiological evaluation intervals (0, 8, 24, and 48 h after inoculation) in the time-kill assays described below.

### Microbiological analysis

Prior to microbiological analyses, the bioglasses were diluted in BHI or Mueller–Hinton (MH) broth, and the suspensions were incubated for 2 h under continuous agitation (100 rpm) in a rotary shaker under aerobic conditions, following a methodology adapted from Drago [13]. Immediately before each dilution step, the suspensions were allowed to settle briefly, and only the supernatant was carefully collected for serial dilutions. In all experiments, growth controls consisted of bacterial suspension in culture medium without bioglass, sterility controls consisted of culture medium only, and negative controls consisted of culture medium supplemented with bioceramic without bacterial inoculum.

### Minimum inhibitory concentration

The minimum inhibitory concentration (MIC) was determined by the broth microdilution method following the guidelines of the Clinical and Laboratory Standards Institute (CLSI, 2020). Briefly, each strain was cultured in BHI broth for 24 h at 37 °C under aerobic conditions. The bacterial suspension was then adjusted to 0.5 McFarland (~ 1.5 × 10⁸ CFU/mL) based on optical density (OD) measured using an Eppendorf BioPhotometer Plus^®^ at 600 nm with a 10 mm path length, and the concentration was confirmed by plating.

Subsequently, the suspension was diluted 1:100 to obtain approximately 1.5 × 10⁶ CFU/mL. A volume of 20 µL of this suspension was inoculated into each well of a 96-well microplate containing 180 µL of a twofold serial dilution of each bioglass formulation prepared in MH broth, resulting in a final bacterial concentration of ~ 1.5 × 10⁵ CFU/mL per well. The tested bioceramic concentrations were 400, 200, 100, 50, 25, 12.5, and 6.25 mg/mL. Growth controls, sterility control and negative control were included. MIC values were defined as the lowest concentration exhibiting no visible bacterial growth after 24 h of incubation at 37 °C under aerobic conditions.

The assay was performed in technical and biological triplicates. MIC results were interpreted using the median value obtained from the microdilution assays. In cases of minor variability among replicates, the median value was adopted, as it represents the most robust measure of central tendency for discrete MIC data.

### Time-kill kinetics

To perform this assay, bacterial strains were streaked onto BHI agar plates and incubated at 37 °C for 24 h under aerobic conditions. Three to five colonies were then transferred to Mueller–Hinton (MH) broth and incubated at 37 °C for 2 h to reach the exponential growth phase. The bacterial suspension was adjusted to 0.5 McFarland turbidity (~ 1.5 × 10⁸ CFU/mL) based on OD measured using an Eppendorf BioPhotometer Plus^®^ at 600 nm with a 10 mm path length, and the concentration was confirmed by plating.

A 10µL aliquot of this suspension was inoculated into test tubes containing 990µL of MH broth, resulting in a final concentration of ~ 1.5 × 10^6^ CFU/mL. The suspensions were supplemented with 45S5 at 400 mg/mL or BVBS at 800 mg/mL, corresponding to 8×MIC for *E. coli* and *P. aeruginosa*, and 4×MIC for *S. pseudintermedius*. Growth controls, sterility control and negative control were included.

The tubes were incubated at 37 °C under aerobic conditions with gentle shaking (100 rpm). Aliquots were collected immediately before bioglass exposure (time 0) and after 8, 24, 48, and 72 h of incubation. Prior to sampling, the suspensions were allowed to settle briefly to minimize particle carryover, and aliquots were carefully collected from the supernatant. Samples were then serially diluted (1:10) in 0.85% saline solution, and 10 µL of each dilution was spotted in technical triplicate onto MH agar plates for viable counts after 24 h of incubation at 37 °C under aerobic conditions. The assay was performed in independent biological triplicates, and the limit of detection of the assay was 1 × 10² CFU/mL.

### Induction of reduced susceptibility

To evaluate the potential induction of reduced susceptibility, each bioglass formulation was prepared at a concentration of 800 mg/mL and pre-incubated under agitation (100 rpm) at 37 °C for 2 h under aerobic conditions. A bacterial inoculum (0.2 mL) adjusted to 3 McFarland (~ 9 × 10⁸ CFU/mL) was added to 1.8 mL of MH broth containing 800 mg/mL of each bioglass, following, partially, the methods described for Drago et al. [13]. The suspensions were incubated at 37 °C under aerobic conditions with agitation (100 rpm) for 48 h.

After 48 h of exposure, 100 µL of the supernatant were collected, subjected to ten-fold (1:10) serial dilutions, and plated onto BHI agar, followed by incubation at 37 °C for 24 h under aerobic conditions. The recovery of viable colonies after this exposure period indicated survival under strong selection pressure. To assess potential induction of reduced susceptibility, a single colony recovered from the BHI plate was selected, and the MIC was re-determined using the broth microdilution method described previously. An increase of at least two-fold in the MIC value compared to the baseline MIC was considered indicative of reduced susceptibility.

## Results

**Fig. 1:**
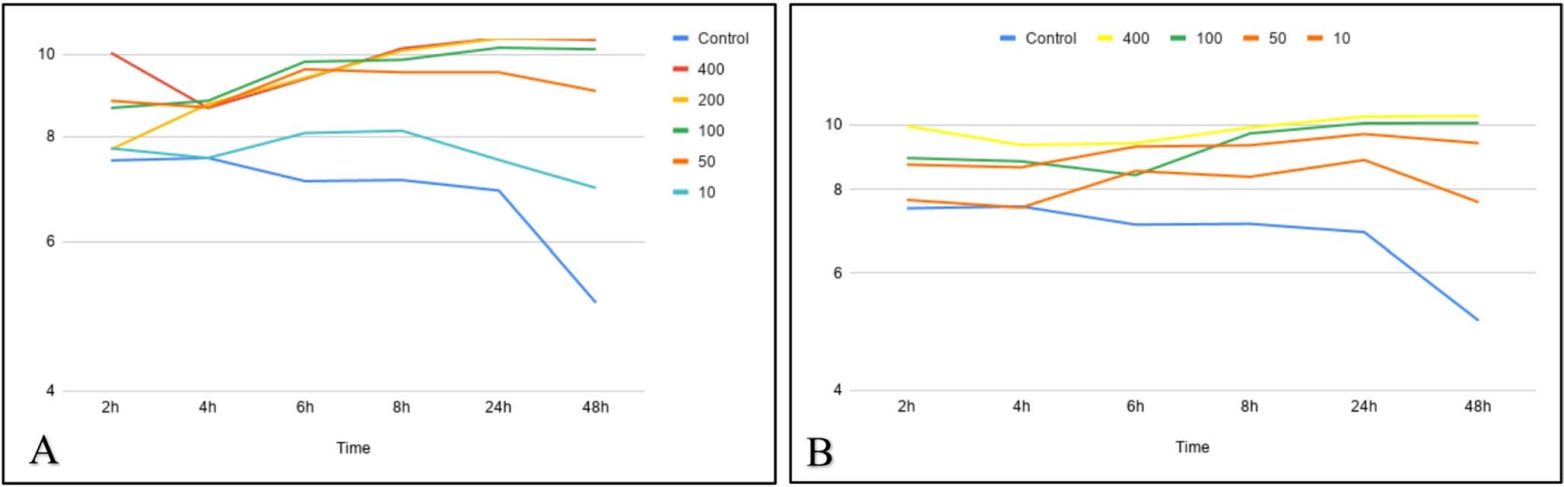
Variation in pH at different concentrations of 45S5 (**A**) and borosilicate (**B**) bioglasses in Mueller–Hinton broth at 2, 4, 6, 8, 24, and 48 hours. Control: measured only in Mueller-Hinton broth

After 6 h of incubation, an increase in pH to values equal to or greater than 8 was observed in all tested concentrations of 45S5 and BVBS (Fig. 1). At concentrations above 50 mg/mL, a peak of alkalinization was observed at 24 h, with pH between 9 and 11, in both formulations. At 48 h, both formulations reached pH greater than 10 at high concentrations (400, 200, and 100 mg/mL). At low concentrations (10 mg/mL), a drop in pH to approximately 7 was observed in both formulations.

**Fig. 2:**
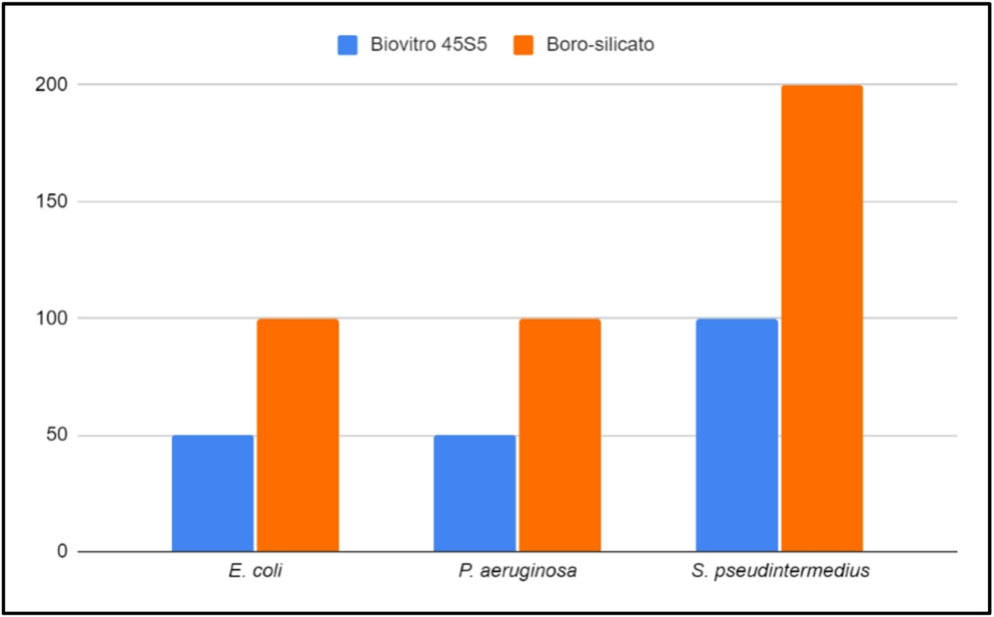
Minimum inhibitory concentrations of 45S5 bioglass and borosilicate bioglass against multidrug-resistant methicillin-resistant *Staphylococcus pseudintermedius*, *E. coli*, and *P. aeruginosa* at concentrations of 50, 100 and 200 mg/mL

The 45S5 and BVBS bioglasses demonstrated the ability to inhibit the growth of *E. coli*, *P. aeruginosa*, and *S. pseudinter*medius strains, as shown in Fig. 2. However, the 45S5 showed greater antimicrobial efficacy, being able to inhibit bacterial growth at lower concentrations when compared to BVBS, in all tests performed. The MIC was lower for the *E. coli* and *P. aeruginosa* strains compared to the *S. pseudintermedius* strain, demonstrating better action against Gram-negative bacteria in both formulations tested (Fig. 2).

**Fig. 3:**
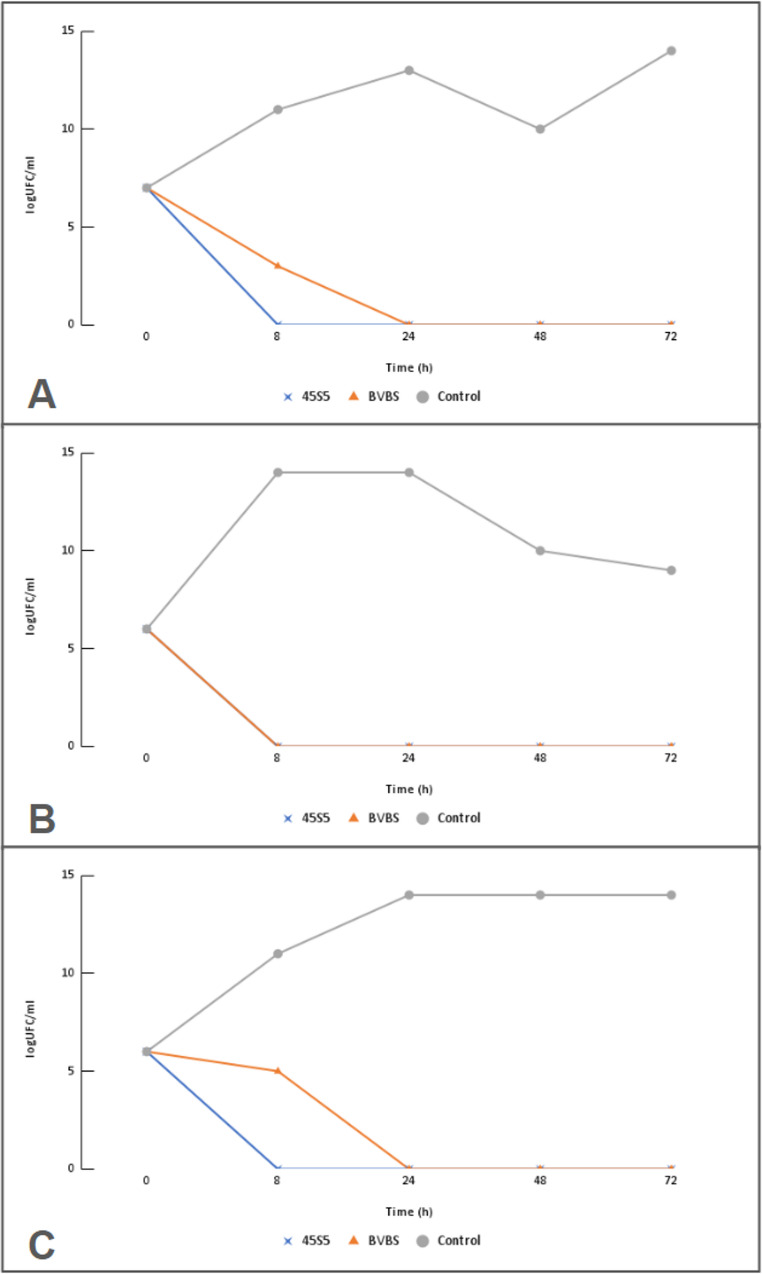
*In vitro* antibacterial activity of 45S5 and borosilicate bioglass (BVBS) against multidrug-resistant bacteria: methicillin-resistant *Staphylococcus pseudintermedius* (**A**), *Escherichia coli* (**B**), and *Pseudomonas aeruginosa* (**C**). Bacterial growth was evaluated at 0, 8, 24, 48, and 72 hours at concentrations of 400 mg/mL for 45S5 and 800 mg/mL for BVBS, corresponding to 4×MIC for *S. pseudintermedius* and 8×MIC for *E. coli* and *P. aeruginosa*

Bioglass 45S5 demonstrated the best bactericidal activity at high concentrations, achieving complete eradication of bacterial growth within 8 h of incubation, regardless of the strain tested (Fig. 3). In contrast, BVBS displayed a delayed antimicrobial response dependent on exposure time, with eradication occurring at 8 h for *E. coli*, 24 h for *P. aeruginosa*, and 72 h for *S. pseudintermedius*.

After the resistance-inducing challenge, no bacterial growth was observed in any of the strains tested with 45S5 bioglass, as well as in the *E. coli* strain treated with BVBS, indicating that no resistance was induced under these conditions. However, BVBS demonstrated the ability to induce resistance in *E. coli* and *P. aeruginosa*, as evidenced by at least a fourfold increase in MIC after the challenge (Table 2).

**Table 2:** Minimum inhibitory concentration before and after induction of reduced susceptibility test for 45S5 and Borosilicate bioglasses against multidrug-resistant methicillin-resistant *Staphylococcus pseudintermedius*, multidrug-resistant *Escherichia coli* and *Pseudomonas aeruginosa*

	45S5		BVBS	
	MIC basal	MIC after IRS	MIC basal	MIC after RI
*E. coli*	50 mg	No growth	100 mg	> 400 mg
*P. aeruginosa*	50 mg	No growth	100 mg	> 400 mg
*S. pseudintermedius*	100 mg	No growth	200 mg	No growth

Legend: 45S5: Bioglass 45S5; VBS: Borosilicate Bioglass; MIC: Minimum Inhibitory Concentration; IRS: Induction of reduced susceptibility

## Discussion

It has been proposed that the antibacterial activity of bioglasses may be associated with local alkalinization resulting from the exchange of sodium ions for protons in aqueous environments, as well as with the release of silicon, calcium, and phosphate ions. These processes have been suggested to affect bacterial membrane potential and increase extracellular osmotic pressure [7, 15–17]. The concentration of solutes in the bacterial cytoplasm is normally higher than that of the external environment, generating positive turgor pressure across the cell membrane [18]. A marked increase in extracellular solute concentration may promote water efflux, reduce transmembrane pressure, and induce alterations in cell morphology and membrane integrity [19].

Additionally, highly alkaline environments may impose significant stress on bacterial cells, potentially leading to changes in morphology, cellular ultrastructure, and gene and protein expression [20, 21]. In the present study, both formulations increased the pH to values between 9 and 11 at concentrations above 50 mg/mL after 8 h of incubation, with a trend toward maximal alkalinization at 24 h. Time–kill kinetic analysis demonstrated complete bacterial eradication at concentrations ≥ 4×MIC after 8 h for 45S5 against all tested strains and for BVBS against *E. coli*, and after 24 h for BVBS against MRSP and *P. aeruginosa*. Although ion release, osmolarity, and ultrastructural alterations were not directly evaluated in this study, the observed bactericidal effect at highly alkaline pH values is consistent with previous reports suggesting that alkalinization may contribute to the antimicrobial activity of bioglasses.

The 45S5 formulation showed greater inhibitory activity, with MICs between 50 and 100 mg/mL, while BVBS showed MICs between 100 and 200 mg/mL, depending on the bacterial species. These findings are consistent with previous studies: Drago [13] and Cordero [22] reported MICs between 100 and 200 mg/mL for S53P4 bioglass against multidrug-resistant strains, and between 50 and 200 mg/mL for 45S5 against *Streptococcus mutans* and *Porphyromonas gingivalis*, respectively. Additionally, both formulations showed greater inhibitory activity (lower MICs) against Gram-negative bacterial species in our study, this trend differs from that reported by Drago et al. [13] and Cordero et al. [22], who observed greater activity against Gram-positive bacteria. However, several methodological differences may account for this variation. These include differences in bioglass composition (S53P4 vs. 45S5 vs. borosilicate formulations), particle size and surface area, bacterial species and strain selection, inoculum density, incubation dynamics (static vs. shaking conditions), exposure time, and culture media composition. As highlighted by Zhang et al. [24], antibacterial performance of bioactive glasses is strongly influenced by experimental conditions that affect ion release kinetics and pH evolution. Therefore, rather than representing a direct contradiction, our findings may reflect differences in experimental design and material characteristics, which can substantially influence MIC values and bactericidal kinetics.

At concentrations ≥ 4xMIC, bacterial eradication was observed between 8 and 24 h, as also described by Drago [13, 23] in similar conditions. However, Zhang [24] observed a bactericidal effect only after 48 h of exposure to S53P4 bioglass in *Staphylococcus epidermidis* using 100 mg/mL, which suggests that efficacy may vary depending on the formulation, the strain tested and the exposure time.

The indiscriminate use of antimicrobials has favored the emergence of multidrug-resistant (MDR) bacteria, making the development of new antimicrobial agents and therapeutic strategies a priority. Compounds with antimicrobial activity can exert selective pressure and contribute to the emergence of resistance [25, 26]. In our study, 45S5 bioglass against all tested strains and BVBS against MRSP did not show reduced susceptibility under the tested conditions, a finding similar to that reported by Drago [13] for S53P4 in similar conditions. This observation is consistent with the hypothesis that the environmental modifications induced by these bioceramics may reduce bacterial viability under the tested conditions.

On the other hand, BVBS demonstrated a potential to induce reduced susceptibility in *E. coli* and *P. aeruginosa* under the tested conditions, with an MIC increase of at least fourfold after prolonged exposure, indicating that Gram-negative bacteria may have greater adaptive capacity to the microenvironment generated by this formulation.

Despite the promising results, this study has some limitations that should be considered. First, the assay was conducted exclusively in vitro, which may not fully reflect the behavior of the bioglasses under clinical conditions, including host factors, vascularization, and immune responses. Second, the evaluation of antimicrobial activity was limited to only three multidrug-resistant bacterial species, which restricts the generalization of the findings to other relevant pathogens involved in bone and surgical site infections. Third, the resistance-induction tests were performed over a short exposure period (48 h), preventing the assessment of potential long-term bacterial adaptations or successive subcultures. Fourth, the absence of a baseline time point (0 h) limits the ability to directly correlate the initial ion-exchange process of the bioglass with the observed pH changes. Finally, the study did not include analyses of biofilm formation, morphological changes, or intracellular damage in the bacteria, which could provide additional insights into the antimicrobial mechanisms of the bioglasses. Further studies are required to better understand the potential of bioglasses to select for bacterial resistance, their effects on bacterial morphology and ultrastructure, possible intracellular damage, and their influence on biofilm formation.

## Conclusion

The results of this study demonstrated that both 45S5 and BVBS bioglasses exhibited in vitro antibacterial activity against multidrug-resistant strains of methicillin-resistant *Staphylococcus pseudintermedius*, *Escherichia coli*, and *Pseudomonas aeruginosa*. The inhibitory effect was concentration-, strain-, and time-dependent. Among the tested formulations, 45S5 showed lower MIC values and more rapid bactericidal activity, achieving complete eradication at ≥ 4×MIC within 8 h for all tested strains. In contrast, BVBS demonstrated a slower inhibitory activity profile, with complete eradication observed at 8 h for *E. coli* and at 24 h for MRSP and *P. aeruginosa* under the same conditions. No increase in MIC was observed for 45S5 or for BVBS against MRSP after 48 h of exposure under the tested conditions. These findings suggest that 45S5, in particular, may represent a promising candidate for further investigation and clinical trials as an adjunct strategy for the control of infections caused by multidrug-resistant bacteria.
